# Information and Efficiency in the Nervous System—A Synthesis

**DOI:** 10.1371/journal.pcbi.1003157

**Published:** 2013-07-25

**Authors:** Biswa Sengupta, Martin B. Stemmler, Karl J. Friston

**Affiliations:** 1 The Wellcome Trust Centre for Neuroimaging, University College London, London, United Kingdom; 2 Centre for Neuroscience, Indian Institute of Science, Bangalore, India; 3 Bernstein Centre Munich, Institute of Neurobiology, Ludwig Maximilians Universität, München, Germany; Indiana University, United States of America

## Abstract

In systems biology, questions concerning the molecular and cellular makeup of an organism are of utmost importance, especially when trying to understand how unreliable components—like genetic circuits, biochemical cascades, and ion channels, among others—enable reliable and adaptive behaviour. The repertoire and speed of biological computations are limited by thermodynamic or metabolic constraints: an example can be found in neurons, where fluctuations in biophysical states limit the information they can encode—with almost 20–60% of the total energy allocated for the brain used for signalling purposes, either via action potentials or by synaptic transmission. Here, we consider the imperatives for neurons to optimise computational and metabolic efficiency, wherein benefits and costs trade-off against each other in the context of self-organised and adaptive behaviour. In particular, we try to link information theoretic (variational) and thermodynamic (Helmholtz) free-energy formulations of neuronal processing and show how they are related in a fundamental way through a complexity minimisation lemma.

## Introduction

The design of engineered and biological systems is influenced by a balance between the energetic costs incurred by their operation and the benefits realised by energy expenditure. This balance is set via trade-offs among various factors, many of which act as constraints. In contrast to engineering systems, it has only been possible recently to experimentally manipulate biological systems—at a cellular level —to study the benefits and costs that interact to determine adaptive fitness [1], [2]. One such example is the nervous system, where metabolic energy consumption constrains the design of brains [3]. In this review paper, we start by defining computation and information in thermodynamic terms and then look at neuronal computations via the free-energy principle. We then consider the efficiency of information processing in the nervous system and how the complexity of information processing and metabolic energy consumption act as constraints. The final section tries to integrate these perspectives: In brief, we will argue that the principle of maximum efficiency applies to both information processing and thermodynamics; such that—for a given level of accuracy—statistically and metabolically efficient brains will penalise the use of complex representations and associated commodities like energy.

## Information Is Physical

A widely used term in neuroscience is “neuronal computation”; but what does computation mean? Simply put, any transformation of information can be regarded as computation, while the transfer of information from a source to a receiver is communication [4]. To understand the physical basis of computation, let us reconsider Feynman's example of a physical system whose information can be read out. The example is intentionally artificial, to keep the physics simple, but has a direct parallel to neuroscience, as we will show at the end. Consider a box that it is filled with an ideal gas containing *N* atoms. This occupies a volume *V*_1_, in which we can ignore forces of attraction or repulsion between the particles. Now suppose that the answer to a question is “yes” if all *N* atoms are on the right-hand side of the box, and “no” if they are on the left. We could use a piston to achieve this. By compressing the gas into a smaller volume *V*_2_, a piston performs the work
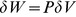
(1)Classical thermodynamics tells us that the pressure and volume of an ideal gas are linked such that
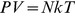
(2)where *k* is Boltzmann's constant and the temperature *T* is assumed constant. The work done on the gas is then:
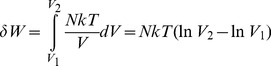
(3)As we compress the gas, the atoms speed up and attain kinetic energy, hence heating the box. According to the conservation of energy, the work done on the gas is converted to heat. This heat is dissipated to the external environment to keep the temperature constant. This means that the internal energy *U* of all the particles remains unchanged, such that the work done by the system or change in *Helmholtz free energy A* = *U*–*TS* reduces to the change in thermodynamic entropy *S* = *kH*, where *H* is Shannon entropy:
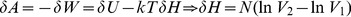
(4)For a single gas particle, with 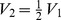 we find that Shannon entropy decreases by ln 2. This means that by compressing the gas there are fewer places that the particles can occupy and we are less uncertain about their whereabouts. In short, we have gained information. What have we learned from this exercise? To obtain information—in other words, to reduce entropy or average uncertainty —one has to perform work. More generally, Landauer's seminal work showed that energy is required when information is erased or deleted via irreversible operations [5], [6]. In the context of noise or communication, the deletion of incorrect bits therefore requires the dissipation of energy. This dissipation is decreased at lower temperatures because of reduced thermal noise—lower temperatures facilitate a reduction of energy expenditure.

In the brain, volume changes are not the primary mode of conveying information. Instead, the compartments present in the brain, ranging from synaptic clefts to organelles, maintain a relatively constant volume over several seconds at least. What changes on a short time scale are the numbers of molecules, such as transmitters or ions, in these compartments. If we translate volumes to concentrations *c_i_* = *N*/*V_i_*, the change in entropy due to information transfer becomes
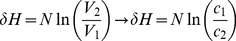
(5)The work is then 
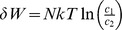
. If the molecules are charged, the chemical potential sets up an electrical potential (called the Nernst potential), which is the basis for much of the signalling within the brain. For some molecules, such as Na^+^ and K^+^ ions, the concentration changes during electrical signalling are miniscule relative to the total concentrations of these molecules. By linearising *δW* in the concentration changes, we can easily compute the energetic cost of neuronal signals [7].

In the examples above, the system remains in thermodynamic equilibrium. Recent progress has been made in describing the relationship between Helmholtz free energy and work when the system is driven far from equilibrium—for example, if the gas was compressed quickly. In this more general setting, the Jarzynski equality states [8]:
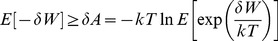
(6)where the expectation *E*[·]is over an ensemble of paths from the initial to final states. Crucially, the change in Helmholtz free energy (and expected work) does not depend upon the path or the rate at which external parameters (like volume) change. Notice that Equation 4 is a special case of Equation 6, when there is only one (infinitely slow) path.

### Summary

In summary, changing the state of a system necessarily entails a change in Helmholtz free energy that is equivalent to the work done on the system. Under isothermal conditions, this changes the thermodynamic entropy, which can be regarded as the average uncertainty or information we have about the (microscopic) state of the system. So is this sufficient to establish the link between thermodynamic free energy and information processing? Not really: because the information here is about the (microscopic) state of the system in question. This does not speak to representational information of the sort associated with biological computations or communication: information of this sort reflects how one system represents another. In the next section, we consider a purely information theoretic perspective on computation that invokes free energy and entropy of a fundamentally different sort.

## The Free-Energy Principle

Equation 4 shows how the basic laws of classical thermodynamics connect the Helmholtz free energy of a system to its entropy, where entropy corresponds to the disorder or average uncertainty about its state. In biological systems, there is a natural tendency to resist disorder—at multiple levels of organisation. The maintenance of sensory and physiological states within characteristic bounds is typical of biological systems and usually relies on some sort of regulatory process, i.e., homeostasis [9], [10]. Mathematically, this can be expressed by saying that the (sensory) states of biological systems have characteristically low Shannon entropy, where—under ergodic assumptions—Shannon entropy is (almost surely) the long-term average of self information or surprise (see below). An ergodic system has an invariant phase volume [11], which is a necessary condition for an organism to exist—in the sense that it would otherwise transgress phase boundaries and cease to exist [12].

Here, the Shannon entropy plays the same role as thermodynamic entropy but measures the dispersion not over microstates of a thermodynamic (canonical) ensemble, but over some phase functions or macroscopic variables that change with time. These variables can take values that are relatively frequent (low surprise) or infrequent (high surprise). Shannon entropy reflects the average surprise of these variables as they fluctuate over time. By minimising the surprise associated with environmental fluctuations (sensory input), an organism can maintain its physiological states within bounds [13], [14].

To evaluate surprise, biological systems need to infer the probability of each sensory fluctuation they encounter. In systems like the brain, these inferences need to be made in the blink of an eye. However, calculating the requisite probabilities can be an intricate and lengthy process, making such computations practically intractable. In 1972, the physicist Richard Feynman came up with a clever trick for calculating probabilities (approximately but very efficiently) using *variational free energy* [15]. The trick is to convert a difficult probability density integration problem into an easy optimisation problem by minimising a free energy bound on the quantity of interest—in our case, the surprise of sensory input. In brief, this entails adjusting probability distributions over the causes of sensory input until they minimise the free energy of sensory input. Notice that we have introduced the notion of causes or hidden states of the world that are responsible for generating sensory samples. Heuristically, this means the system or agent has a model of the world that it uses to evaluate the likelihood or surprise of a sensation. Mathematically, hidden states are fictive variables that are necessary to construct a variational free energy bound on surprise, as we will see next.

Let us assume that self-organising systems like the brain represent their environment probabilistically, in terms of hidden states that cause sensory input. For example, an agent might believe its visual sensations were caused by a bird flying across its field of view. These beliefs can be regarded as real-valued, time-dependent internal or representational states 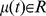. These internal states encode a conditional probability density 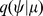 over hidden states in the world 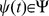
—such as the motion, colour, and size of the bird. The objective is to minimise the surprise 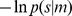 of sensations 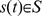. Here, 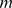 denotes a model entailed by a system or an agent, and 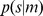 is the probability of observing a particular state under that model. The model is effectively used to generate hypotheses that explain sensory input in terms of hidden states or representations—such as a bird in flight.

As noted above, minimising surprise directly is an intractable problem, so surprise is replaced with its variational free energy bound [15]. This free energy is a function of sensory and internal states and can now be minimised with respect to the internal states:
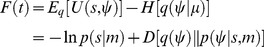
(7)Here, *U*(*t*) = −ln *p*(*s*(*t*), *ψ*(*t*)|*m*) corresponds to an internal energy under a generative model of the world, described in terms of the density over sensory and hidden states *p*(*s*,*ψ*|*m*). In Equation 7 and throughout *H*[*p*] = *E_p_*[−ln *p*] denotes the entropy of a probability distribution. Comparison with Equation 4 explains why *F*(*t*) is called free energy—by analogy with its thermodynamic homologue that is defined as internal energy minus entropy. However, it is important to note that variational free energy is not the Helmholtz free energy in Equation 4—it is a functional of a probability distribution over hidden (fictive) states *encoded by* internal states *q*(*ψ*|*μ*), not the probability distribution over the (physical) internal states. This is why variational free energy pertains to information about hidden states that are represented, not the internal states that represent them. In other words, the variational free energy measures the information represented by internal states, not internal states *per se*. Later, we will try to establish the link between variational and Helmholtz free energies. First, we consider the computational implications of minimising variational free energy.

In short, free energy finesses the evaluation of surprise—where an agent can evaluate free energy fairly easily, given the internal energy or a generative model of its environment. The second equality in Equation 7 says that free energy is always greater than surprise, because the second term (Kullback-Leibler divergence) is nonnegative. This means that when free energy is minimised with respect to the internal states, free energy approximates surprise and the conditional density approximates the posterior density over hidden states:
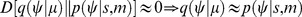
(8)This is known as approximate Bayesian inference, which becomes exact when the conditional and posterior densities have the same form [16]. Intuitively, minimising free energy renders the conditional density the true posterior density over hidden states, where both are informed by—or conditioned on—sensory information. In Bayesian parlance, a posterior density describes a belief after sampling some data—in contrast to a prior belief that existed before the data were available. Minimising variational free energy can therefore be regarded using sensory evidence to update prior beliefs to approximate posterior beliefs.

How can we place a concept like variational free energy in the context of neuronal computation? This has a long history—originating in Geoffrey Hinton [17], [18] and Douglas Hofstadter's [19] work using Ising models for inference in artificial neural networks. Hinton and colleagues realised that variational free energy was mathematically equivalent to the cost function for inference in a neural network, such as a Hopfield model [20]—the difference between the prediction made by the neural network and what it actually produced as an output, i.e., the prediction error. These ideas were subsequently absorbed into the free-energy principle [21], [22], whose key insight was that to reduce the entropy of sensations, the system had to act on the environment. The solution is to assume that both the internal states of the system and its action minimise variational free energy (and implicitly surprise). This dual minimisation maps nicely onto perception and action, where variational free energy can be reduced by optimising internal (representational) states or sensory states through active sensory sampling. This is known as active inference and essentially compels organisms to selectively sample what they expect to sample.

Under certain statistical assumptions, free energy is essentially the difference between the agent's predictions and the actual sensations sampled [22]. Therefore, minimising the free energy is equivalent to reducing prediction error and hence surprise [14]. To minimise free energy or prediction error, the brain can either change its prediction to match sensory input or it can change what it samples to match its predictions [21]. This suggests that the brain is continually making predictions and reevaluating them by comparing inputs with internal predictions to make sense of the world. Is there any empirical evidence that this scheme operates in the nervous system?

Volunteers in a magnetic resonance imaging (MRI) scanner watched two sets of moving dots—one random and the other moving coherently. They showed patterns of distributed brain activation that could only be explained in terms of top-down predictions from deep in the brain to visual centres in the occipital cortex. In other words, top-down predictions from the extrastriate cortex appeared to suppress prediction errors in the striate cortex [23]. Assuming the visual system is a hierarchy of cortical areas, such predictive coding enables predictions about hidden states of the world—like coherent motion—to influence processing at lower levels [23]. Similarly, in the auditory cortex, electroencephalographic signals from higher processing centres change brain activity in lower areas [24]. Using dynamic causal modelling, Garrido *et al.* [24] found that models with top-down connections explained empirical electrophysiological data far better than the models with only bottom-up connections. Garrido *et al.* [24] argued that these neuronal responses were consistent with the brain's attempt to conciliate predictions at one level with those in other levels—in other words, to reduce hierarchical prediction error.

What sort of neuronal architectures mediate this prediction error minimisation—or predictive coding? In mammalian brains, cortical areas are organised hierarchically [25], [26], wherein populations of neurons can encode expected states of the world and provide top-down predictions to lower or sensory levels [27], [28]. For example, top-down connections from pyramidal neurons in the deeper layers of the cortex are thought to provide predictions to superficial pyramidal populations of a lower area. This enables forward connections from superficial pyramidal neurons to convey prediction errors, creating recurrent dynamics that suppress prediction errors at each level of the cortical hierarchy [29]–[31]. The precision of these errors can be modulated by neuromodulation [32]. Such rescaling of prediction errors in proportion to their precision is simply a form of gain control [33], [34] and may mediate attention. In short, the wetware necessary to minimise free energy appears to be available and is remarkably consistent with its known functional anatomy.

In summary, biological organisms are open self-organising systems that operate far from thermodynamic equilibrium [35]. The free-energy principle suggests that organisms avoid phase transitions by minimising (a variational free energy bound on) the Shannon entropy of their sensory states. But how does one reconcile the need of an animal to survive (by avoiding phase transitions) with its innate tendency to forage or explore? This apparent paradox is resolved by noting that active inference is driven by prior beliefs—and these beliefs can entail exploration. In other words, agents expect to explore and would be surprised if they did not. We will return to the central role of priors in the last section.

### Summary

Perception minimises prediction error by optimising synaptic activity (perceptual inference), synaptic efficacy (learning and memory), and synaptic gain (attention and salience) [14]. In doing so, we form an optimal representation of the sensorium. Such strategies of optimisation are mathematically equivalent to predictive coding [36], [37] or, as we will see later, maximising the mutual information between sensations and the responses they evoke [38], [39]. In the embodied context of action on the environment, free-energy minimisation can also explain active inference in the exteroceptive domain [40] and homoeostasis through minimising interoceptive prediction errors. In short, the idea of free-energy minimisation, stemming from Feynman's beautiful piece of mathematics, allows us to consider perception and action under a general framework—and produce testable hypotheses.

## Information Efficiency

In the previous section, we described how variational free energy is intricately linked to surprise—the free-energy principle tells us that an organism should strive to reduce its prediction error thereby reducing free energy. The connection between free energy and information—although obvious—is seldom commented upon (see Table 1 in [41]). To minimise free energy, the expected prediction error has to be minimised while, at the same time, the entropy of the conditional density is maximised. This is slightly paradoxical because the purpose of free-energy minimisation is to reduce sensory entropy. However, Equation 7 shows that if the entropy of sensory states *H*[*p*(*s*|*m*)] is minimised vicariously by minimising free energy over time, then the entropy of the conditional density *H*[*q*(*ψ*|*μ*)]must be maximised at each point in time. This follows from a need to balance accuracy and complexity of the sort seen in Occam's razor. We will return to this in a later section in the context of the principle of maximum entropy [42]. In this section, we focus on information theory as a way of describing the quality of representations and the constraints under which these representations are formed.

We know that all animals process and transmit information to survive and reproduce in an uncertain environment. A principled way to understand such signal processing was absent until Claude Shannon's seminal work on information theory [43]. To understand how messages can be transferred efficiently via telegraphic wires, Shannon derived powerful formalisms that provided fundamental limits on communication [43]. On one hand, information theory allowed optimisation of complicated devices like satellite communication systems. On the other hand, it fitted comfortably with the bounds established by thermodynamics [44]. Some years after its inception, biologists used information theory to study the efficiency of processing in the nervous system. It was realised that efficient representations were permitted by statistical regularities in the sensorium, i.e., hidden states and their sensory consequences that have low entropy (see [45]). However, the influence of random fluctuations and other constraints prohibit a completely efficient encoding of hidden states in the world.

In the nervous system, limited bandwidth and dynamic range create an information bottleneck due to the limited response ranges of the neurons in sensory epithelia [46]–[48]. Atick [45] suggests that these bottlenecks can also result from computational limitations at higher levels of sensory processing—citing as an example the “attention bottleneck,” where there is constriction of information processing—in bits per unit time—somewhere between area V4 and the inferotemporal cortex. In brief, sensory receptors are required to compress an enormous range of statistically redundant sensory data into their limited range. One way to achieve this is by compression —imagine an architect's plan of your office. This does not include the dimensions of every brick, just the information necessary to build the office. It has been proposed that sensory systems also apply the principle of compression. They sieve redundant information, such that only information that is necessary to encode hidden states is retained [46]—in engineering this is called a factorial code. Of course there are many ways to describe such sensory encoding. Others include but are not restricted to feature detection, filtering, etc. Among these, schemes like linear predictive coding and minimum description length formulations have a particularly close and formal relationship with variational formulations.

**Figure 1:**
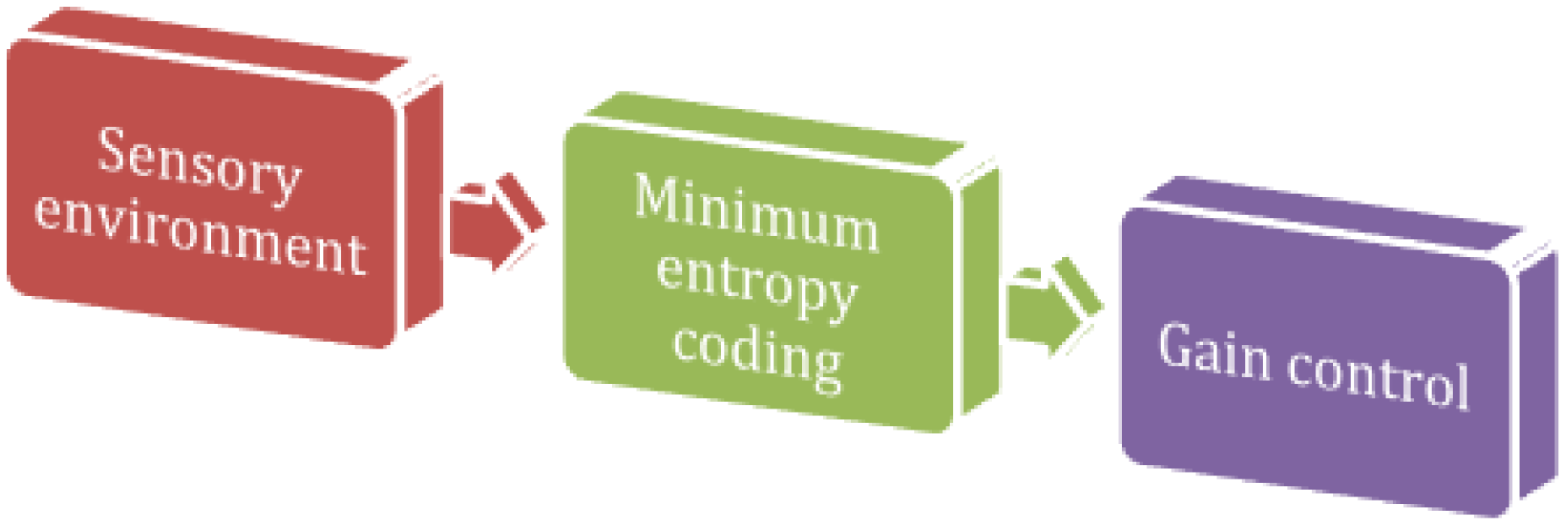
Redundancy reduction. The sensory environment of an animal is highly correlated (redundant). The animal's job is to map such signals as efficiently as possible to its neuronal representations, which are limited by their dynamic range. One way to solve this problem rests on de-correlating the input to provide a minimum entropy description, followed by a gain controller. This form of sensory processing has been observed in the experiments by Laughlin [49], where the circuit maps the de-correlated signal via its cumulative probability distribution to a neuronal response, thereby avoiding saturation. Modified from [45].

Sensory receptors (mechanoreceptors, photoreceptors, and the like) are thought to build a factorial representation of the world—such that only independent bits of information are sampled (Figure 1). Interestingly, this has been observed in the large monopolar cells (LMC) in the blowfly compound eye [49]. Laughlin [49] measured the distribution of the fly's natural environment from horizontal scans of dry woodland and lake-side vegetation and quantified the responses of light-adapted LMCs. Laughlin [49] found that the LMC—known to respond to contrast signals—is most sensitive around the most probable input contrast—with sensitivity dropping to zero as the input became more improbable.

The application of information theory to the nervous system is formally pleasing and has provided some compelling insights. However, it does have limits [50]: although it allows one to quantify the transmission of information, it has no notion of semantics. It only cares about how much information is present but not about what that information represents. A widely used information theoretic metric in neuroscience is the mutual information, which measures how much a random variable tells us about another random variable [51]. If 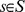 is a stimulus and 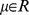 is the representational response, the mutual information is defined as:
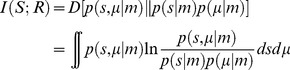
(9)Note that the joint density *p*(*s*,*μ*|*m*) is not the generative model *p*(*s*,*ψ*|*m*) of the previous section—it describes the joint distribution of sensory and internal states, not the joint distribution of sensory and hidden states. Equation 9 simply describes the divergence or relative entropy between the joint density and the product of its marginals. The mutual information is zero when the neuronal representation is statistically independent of the stimulus and is equal to the entropy of the stimulus when the representation faithfully encodes the stimulus. Since the mutual information must lie between zero and channel capacity, it is only the channel capacity that limits the information transfer between stimulus and neuronal response.

Estimating channel capacity by maximising empirical estimates of mutual information can be a difficult task, especially when the experimenter has only an informed guess about the stimuli that evoke responses. One way to finesse this problem is to use adaptive sampling of inputs, which hones in on stimuli that are maximally informative about observed responses [52]. Assuming one knows the stimuli to use, the next problem is the curse of dimensionality. In other words, one requires an enormous amount of data to estimate the probability densities required to quantify mutual information. Although, sophisticated machine learning tools try to estimate mutual information from limited data [53]–[55], the numerics of mutual information are fraught with difficulties.

### Summary

Irrespective of the thermodynamic or computational imperatives for a biological system, the simple observation that there should be some statistical dependency between sensory samples and the internal states that encode them means that sensory and internal states should have a high mutual information. This leads to the principles of maximum information transfer (a.k.a. infomax) and related principles of minimum redundancy and maximum efficiency [46]–[48]. Later, we will see how minimising variational free energy maximises mutual information and what this implies for metabolic costs in terms of Helmholtz free energy. First, we will briefly review the biophysical and metabolic constraints on the information processing that underlies active inference.

## Is Inference Costly?

Hitherto, we have considered the strategies that neurons might use for abstracting information from the sensorium. A reliable representation is necessary for an animal to make decisions and act. Such information processing comes at a price, irrespective of whether the animal is at rest or not [56]. Cellular respiration enables an organism to liberate the energy stored in the chemical bonds of glucose (via pyruvate)—the energy in glucose is used to produce ATP. Approximately 90% of mammalian oxygen consumption is mitochondrial, of which approximately 20% is uncoupled by the mitochondrial proton leak and 80% is coupled to ATP synthesis [57]. Cells use ATP for cellular maintenance and signalling purposes, via ion channels that use ATP hydrolysis to transport protons against the electromotive force. Given that the biophysical “cash-register” of a cell (the ATPases) can only handle ATP—and not glucose—we will discuss brain metabolism in terms of ATP.

**Figure 2:**
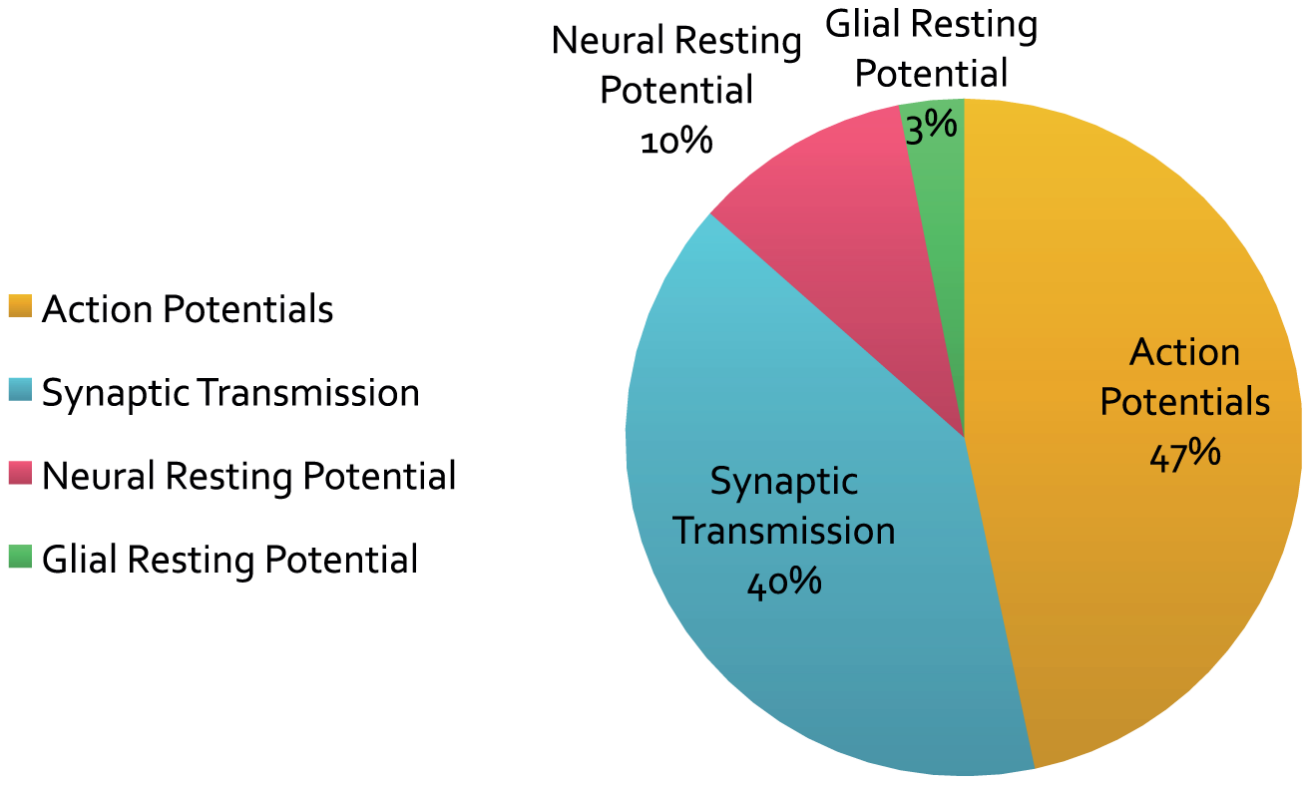
Attwell and Laughlin's energy budget. Energy use by various neuronal (cellular) processes that produce, on average, 4 spikes per second. Modified from [59].

In man, the brain constitutes just 2% of the body mass, while consuming approximately 20% of the body's energy expenditure for housekeeping functions like protein synthesis, maintenance of membrane potentials, etc. [58]. What consumes such remarkable amounts of energy? Assuming a mean action potential (AP) rate of 4 Hz, a comprehensive breakdown of signalling costs suggests that action potentials use around 47% of the energy consumed—mainly to drive the Na^+^/K^+^ pump (Figure 2) [59]. This pump actively pumps Na^+^ ions out of the neuron and K^+^ ions inside [60]. In doing so, the pump consumes a single ATP molecule for transporting three Na^+^ ions out and two K^+^ ions in [61]–[63]. Measurements of ATP consumption from intracellular recordings in fly photoreceptors show similar energy consumption to costs obtained from whole retina oxygen consumption [64], [65]. Indeed, in the absence of signalling, the dominant cost of maintaining the resting potential is attributable to the Na^+^/K^+^ pump. Attwell and Laughlin [59] further estimated that out of 3.29×10^9^ ATP/s consumed by a neuron with a mean firing rate of 4 Hz, 47% was distributed for producing APs, while postsynaptic receptors accounted for around 40% of the energy consumption (Figure 2). These figures suggest that action potentials and synapses are the main consumers of energy and that they determine the energy cost in the nervous system.

Experimental studies have shown that neuronal performance is related to energy consumption, both during rest and while signalling [65]. What these studies show is obvious—there is no free lunch. Neurons have to invest metabolic energy to process information. The finite availability of ATP and the heavy demand of neuronal activity suggest neuronal processing has enjoyed great selective pressure. Metabolic energy costs limit not only the possible behavioural repertoire but also the structure and function of many organs, including the brain [3], [66], [67]. The nervous system can use many tricks to promote energy efficiency. Neurons that use sparse (or factorial) codes for communication [48], [68] save on the number of action potentials required to encode information, or have topographical connectivity schemes to reduce the surface area of axons connecting different brain areas [69]–[71]. Neurons may also alter their receptor characteristics to match the probability of inputs to form a matched filter [49]. Alternatively, specialised signal processing could be employed to convert signals from analogue representation to pulsatile—prohibiting accumulation of noise during information transfer [72], [73].

**Figure 3:**
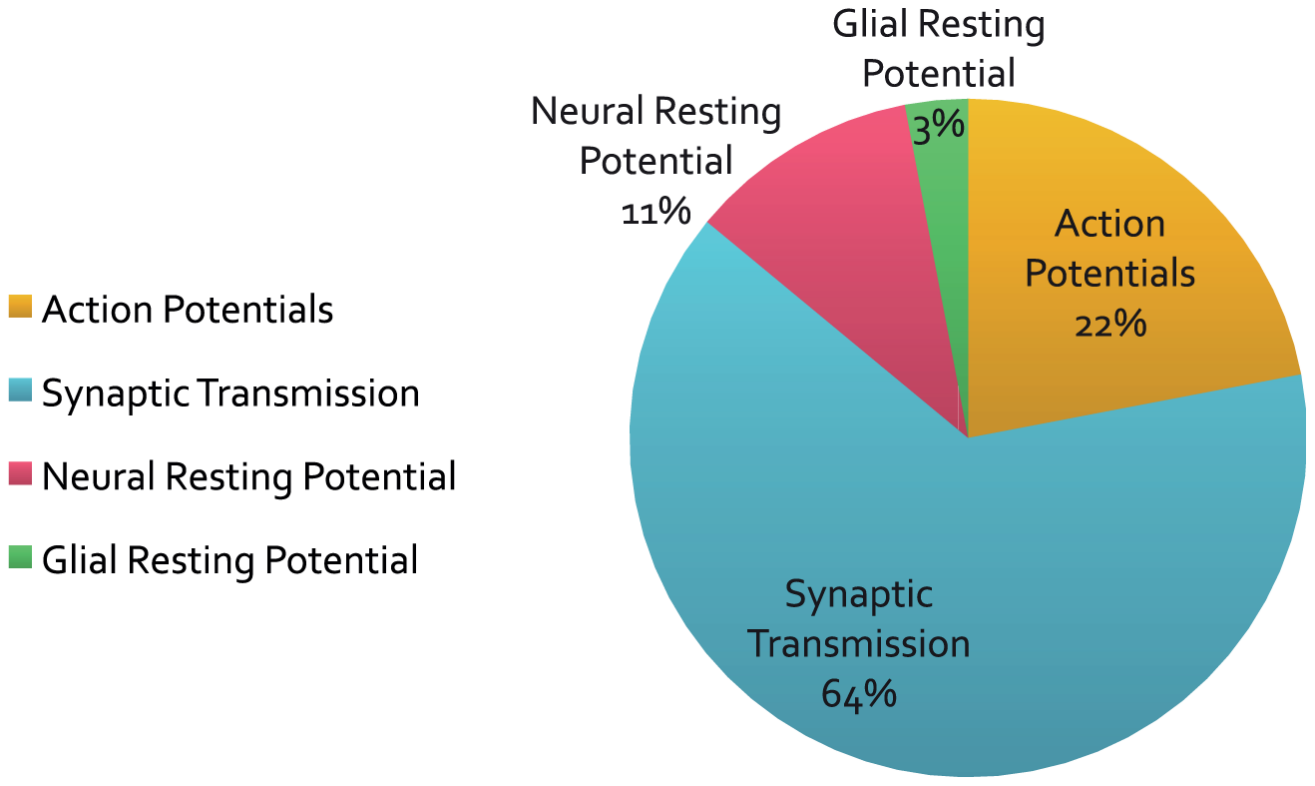
A revised energy budget for signalling in the grey matter of the rat brain. Incorporating the increased efficiency of APs in mammalian neurons into Attwell and Laughlin's (Figure 2) original energy budget—for grey matter in the rat brain—reduces the proportion of the energy budget consumed by APs. Modified from [80].

In short, nature can use various means to achieve the objective of energy efficiency—see Box 1 for a summary of some strategies. Energy consumption in single neurons depends on the types and the numbers of ion-channels expressed on the lipid bilayer, their kinetics, the cell's size, and the external milieu that changes the equilibrium conditions of the cell. Experimental measures from the blowfly retina show that metabolic efficiency in graded potentials (lacking voltage-gated Na^+^ channels) is at least as expensive as in those neurons displaying action potentials—with the former capable of higher transmission rates [74]. Similarly, in *Drosophila melanogaster* photoreceptors, absence of Shaker K^+^ conductance increases energetic costs by almost two-fold [75], [76]. It has also been suggested that the precise mix of synaptic receptors (AMPA, NMDA, mGlu, Kainate, etc.)—that determine synaptic time constants—influences the energetic cost of the single neuron [77]. Recent evidence indicates that the biophysical properties generating an action potential can be matched to make them energy efficient [78]–[81]. Fast Na^+^ current decay and delayed K^+^ current onset during APs in nonmyelinated mossy fibres in the rat hippocampus minimise the overlap between the inward and outward currents, resulting in a reduction of metabolic costs [81]. Similarly, incomplete Na^+^ channel inactivation in fast-spiking GABAergic neurons during the falling phase of the AP reduces metabolic efficiency of these neurons [78]. Applying numerical optimisation to published data from a disparate range of APs, Sengupta *et al.* [80] showed that there is no direct relationship between size and shape of APs and their energy consumption. This study further established that the temporal profile of the currents underlying APs of some mammalian neurons are nearly perfectly matched to the optimised properties of ionic conductances, so as to minimise the ATP cost. All of these studies show that experimentally measured APs are in fact more efficient than suggested by the previous estimates of Attwell and Laughlin [59]. This was because until 2001 experimental measurements of membrane currents were scant, impeding the study of the overlap between Na^+^ and K^+^ currents. The effects of energy-efficient APs on cortical processing were gauged by recalculating Attwell and Laughlin's (2001) estimates by first using the overlap factor of 1.2—found in mouse cortical pyramidal cells—and then assuming the probability that a synaptic bouton releases a vesicle in response to an incoming spike remains at 0.25 [80]. Neurons that are 80% efficient have two notable effects (Figure 3). First of all, the specific metabolic rate of the cortical grey matter increases by 60%, and second, the balance of expenditure shifts from action potentials to synapses (Figure 3, *cf.* Figure 2) [80].

**Box 1. Some principles of computational anatomy**

**Dimensionality reduction:** Sensory input is high dimensional—a visual scene comprises differences in brightness, colours, numbers of edges, etc. If the retina did not preprocess this visual information, we would have to handle around 36 Gb/s of broadband information, instead of 20 Mb/s of useful data [73]. Preprocessing increases the metabolic efficiency of the brain by about 1,500 times. The requisite dimensionality reduction is closely related to minimising complexity—it is self-evident that internal representations or models of the sensorium that use a small number of dimensions or hidden states will have a lower complexity and incur smaller metabolic costs.

**Energy-efficient signalling:** Action potentials (APs) are expensive commodities, whether they are used for local computation or long-distance communication [59]. Energy-efficient APs are characterised by Na^+^ channel inactivation, voltage-dependent channel kinetics, and corporative K^+^ channels—as described by multiple gating currents, inward-rectifying K^+^ channels, and high channel densities [7]. These biophysical innovations enable a neuron to produce efficient APs that use the minimal currents necessary to generate a given depolarisation.

**Component size and numbers:** Action potentials travel considerable distances along densely packed axons, collaterals, and dendrites. The capacitance that must be charged by APs increases with membrane area [101], constraining the number and length of neuronal processes. It is fairly straightforward to show that—to maintain information transfer—the optimal solution is to decrease the number of components. Assuming all neurons have the same thresholds and energy consumption, the energy-efficient solution is to minimise the number of components, under computational constraints dictated by the ecological niche of the animal [101].

**Modular design:** Very-large-scale integration circuits suggest an isometric scaling relation between the number of processing elements and the number of connections (Rent's rule [102]). Neuronal networks have been shown to obey Rent's rule, exhibiting hierarchical modularity that optimises a trade-off between physical cost and topological complexity—wherein these networks are cost-efficiently wired [103]. A modular design balances the savings in metabolic costs, while preserving computational capacities. Hierarchical modularity also emerges under predictive coding [33]. In this context, the brain becomes a model of its environment, which through the separation of temporal scales necessarily requires a hierarchical connectivity.

**Parallel architecture:** The brain processes information in parallel—be it frequency analysis in the inner ear or analysing different attributes of a visual scene using functional segregation. This parallel architecture mirrors those used in modern-day microprocessors. For example, a fast single-core microprocessor may consume 5 Watts and execute a program in 10 seconds. If we bring together two single cores, power will double and execution time will halve, still consuming 50 Joules. Alternatively, a slow double-core microprocessor that expends 2.5 Watts of power to execute the program in 15 seconds could consume only 7.5 Joules. This energy saving works because power is proportional to frequency cubed; therefore, halving the frequency reduces the speed by two but conserves eight times the power, making the microprocessor four times as efficient. In short, if parallel architectures are combined with slow computing speeds, the resulting system is energetically more efficient.

**Analogue versus digital:** If analogue computing is so efficient [104], why don't neurons operate on an all analogue basis? The obvious answer is signal processing in the digital (such as AP) domain enables noise suppression. Noise accumulation in analogue systems [73] speaks to hybrid processing—the use of analogue preprocessing before optimal digitisation. APs are useful in this context because they have an inbuilt threshold mechanism that attenuates noise. If a presynaptic signal is encoded as an AP and transmitted, there is hardly any conduction loss, thereby enabling a reliable transfer of information.

**Figure 4:**
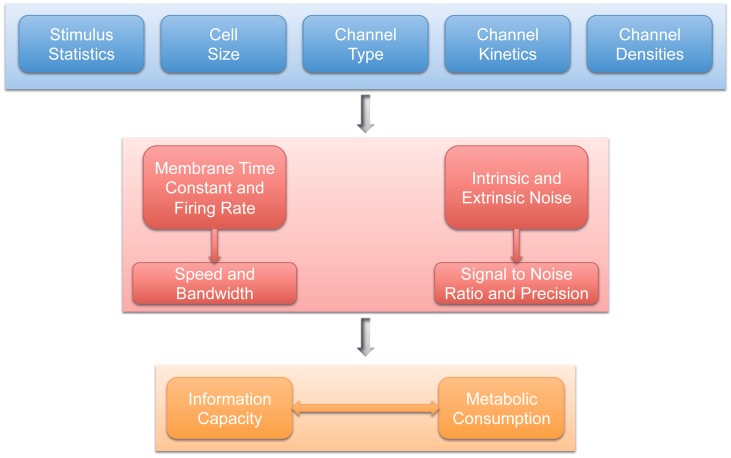
Elements defining metabolic efficiency. Speed and precision defines the representational capacity of a neuron. Speed or bandwidth is dependent on the membrane time constant and/or the spike rate of the neuron, while precision relies mainly on the types, numbers, and kinetics of synapses and the channels, neuron volume, etc. An efficient brain will maximise speed and precision under energetic constraints.

The principle of energy efficiency is not just linked to single neurons. Energy budgets have been calculated for the cortex [82], olfactory glomerulus [83], rod photoreceptors [84], cerebellum [85], and CNS white matter [86], among others. These studies highlight the fact that the movement of ions across the cell membrane is a dominant cost, defined by the numbers and cellular makeup of the neurons and the proportion of synaptic machinery embedded in the cell membrane (Figure 4). Niven and Laughlin [3] have argued that when signalling costs are high and resting costs are low, representations will be sparse; such that neurons in a population preferentially represent single nonoverlapping events (also see [87]). Similarly, when resting costs are high and signalling costs are low, the nervous system will favour the formation of denser codes, where greater numbers of neurons within the population are necessary to represent events [3].

Experimental studies of mammalian cortex suggest that the cortex organises itself to minimise total wiring length, while maximising various connectivity metrics [88]. Minimising wiring lengths decreases the surface area of neuronal processes, reducing the energy required for charging the capacitive cell membrane—to sustain and propagate action potentials. In fact, theoretical analyses in pyramidal and Purkinje cells have shown that the dimensions and branching structure of dendritic arbours in these neurons can be explained by minimising the dendritic cost for a potential synaptic connectivity [89], [90]. This can result from increasing the repertoire of possible connectivity patterns among different dendrites, while keeping the metabolic cost low [89], [90].

### Summary

In summary, we have reviewed several lines of evidence that evolution tries to minimise metabolic costs, where—in the brain—these costs are primarily incurred by the restoration of transmembrane potentials, whose fluctuations encode or represent hidden states of the world. This raises a question: is energy the only constraint in the evolution of animals? Of course not—functional constraints like reliability, speed, precision, etc. [67] and structural constraints like optimal wiring [91] are equally important. For example, a single action potential in the squid giant axon consumes orders of magnitude more energy than a hippocampal or a pyramidal neuron, yet evolution has invested that extra Joule to buy speed [80], [92]. In short, structure and function interact to determine the fitness of an animal. Having surveyed the key metabolic constraints under which neuronal processing must proceed, we now try to integrate the information theoretic and metabolic perspectives.

## Thermodynamic Efficiency and Free-Energy Minimisation

In this section, we gather together the imperatives for biological self-organisation reviewed above. We hope to show that minimising variational free energy necessarily entails a metabolically efficient encoding that is consistent with the principles of minimum redundancy and maximum information transfer. In brief, we will show that maximising mutual information and minimising metabolic costs are two sides of the same coin: by decomposing variational free energy into accuracy and complexity, one can derive the principle of maximum mutual information as a special case of maximising accuracy, while minimising complexity translates into minimising metabolic costs.

### Metabolic Efficiency and Free Energy

To connect the thermodynamic work or metabolic energy required to represent hidden states to the variational free energy of those representations, we need to consider the relationship between representational internal states and the underlying thermodynamic microstates. Recall that internal states *μ*(*t*) are deterministic quantities that encode a conditional density over hidden states of the world. These macroscopic states can be regarded as *unconstrained internal variables* of a biophysical system; for example, the molar fractions of different molecules in a cellular compartment. The underlying biophysical system can then be associated with a (thermodynamic) canonical ensemble with internal energy:
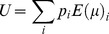
(10)Here, *p_i_* corresponds to the probability of a particular microscopic state and *E_i_*(*μ*)to its corresponding energy. Given that the total energy is conserved, this probability is given by the Gibbs measure or Boltzmann distribution:
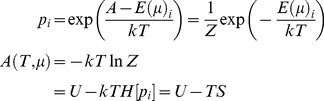
(11)The partition function *Z*(*T*, *μ*) ensures the probabilities sum to one, while the last equality follows simply from the definition of entropy *H*[*p_i_*] = *E_i_*[−ln *p_i_*]. The Boltzmann distribution describes a system that can exchange energy with a heat bath (or a large number of similar systems) so that its temperature remains constant. The Helmholtz free energy *A*(*T*, *μ*) measures the work obtainable from a closed thermodynamic system at a constant temperature and volume—where a closed system can exchange energy with other systems (but not mass).

The key result we will use from statistical thermodynamics is that the Helmholtz free energy is minimised at equilibrium with respect to any unconstrained internal variables for a closed system at constant temperature *T*_0_,
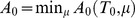
(12)where *A*_0_(*T*_0_, *μ*) is the free energy of the system at equilibrium or steady state (i.e., constant entropy). This motivates the following Lemma:

**Lemma**: *(complexity minimisation) Minimising the complexity of a conditional distribution—whose sufficient statistics are (strictly increasing functions of) some unconstrained internal variables of a thermodynamic system—minimises the Helmholtz free energy of that system.*

**Proof**: Using standard results from Bayesian statistics [16], we can express free energy as *complexity* minus *accuracy*

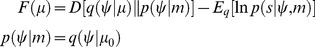
(13)The first complexity term is the divergence between the conditional distribution and the prior distribution under the generative model. This effectively counts the degrees of freedom used to encode or predict sensory input. The accuracy is simply the expected log likelihood of the sensory input under the conditional density encoded by internal states. The prior distribution represents beliefs in the absence of sensory input. This corresponds to the distribution encoded by internal states *μ* = *μ*_0_ when deprived of input for a suitably long time—at which point, we can assume thermodynamic equilibrium, such that Helmholtz free energy is minimised (see Equation 12):
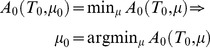
(14)However, in the absence of input, variational free energy reduces to complexity *F*_0_(*μ*)≥0, which—by Gibbs inequality—has a minimum of zero. This means that complexity is also minimised.
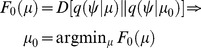
(15)In sum, the internal states encoding prior beliefs about hidden states of the world are those that minimise Helmholtz free energy and the complexity defined by variational free energy.

**Remarks**: All we are saying here is that if a (neuronal) system is deprived of sensory inputs it will obtain thermodynamic equilibrium (or at least a nonequilibrium steady state) and will therefore minimise Helmholtz free energy. This assumes, not implausibly, a constant temperature and volume. Crucially, this is precisely the brain state encoding prior beliefs about sensory input, which means that it is the state of minimum computational complexity. Heuristically, this means that one can associate the complexity cost of variational free energy with metabolic cost—in the sense that they share the same minimum. Crucially, minimising fluctuations in Helmholtz free energy reduces metabolic work by Equation 6. Interestingly, complexity cost also plays a central role in free-energy formulations of optimal control and economic theory [93], [94]. Still *et al.* arrive at the same conclusions by treating the thermodynamic system as having an implicit model of its inputs—allowing them to establish the fundamental equivalence between model inefficiency or complexity and thermodynamic inefficiency [95]. However, both of these compelling treatments consider homologues of Helmholtz free energy—not variational free energy, which is a functional of a probabilistic model (the conditional distribution).

### Computational Efficiency and Free Energy

The complexity minimisation lemma suggests that commonly occurring representational states—that are *a priori* most probable—are the least costly; for example, resting levels of transmembrane voltage or baseline firing rates. Rare excursions from these states are associated with a high metabolic cost. But how does minimising complexity relate to principles of minimum redundancy? Because representations do not change sensory inputs, they are only required to minimise the free energy of the conditional density. Assuming conditional uncertainty is small, the conditional density can be approximated with a point mass at 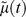, such that 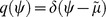 and the free energy becomes (from Equation 13)
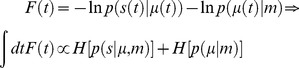
(16)The first equality expresses free energy is terms of accuracy and complexity, where the second complexity term just reports the surprise about the conditional representation under prior beliefs. The second equality is the corresponding path integral of free energy (known as free action). Under ergodic assumptions [12], [96] this can be expressed as the conditional entropy of sensory input, given the representations and the entropy of the internal states. Equation 11 has two important implications. First, it shows that minimising free energy, at each point in time, is equivalent to minimising free action —by the fundamental lemma of variational calculus. In other words, Equation 11 is just a restatement of the principle of least action. Second, it shows that minimising free energy maximises the accuracy of representations or minimises their conditional uncertainty (entropy) over time. This is simply a restatement of the principle of minimum redundancy or maximum mutual information [97]. This follows because minimising uncertainty about sensory inputs, given internal states, implicitly maximises the mutual information between sensory and internal states (for any given sensations):
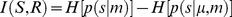
(17)This suggests that the infomax principle [97] is a special case of the free-energy principle that is obtained when we discount uncertainty and represent sensory input with point estimates of their causes. In this context, high mutual information is assured by maximising accuracy (e.g., minimising prediction error) and prior beliefs are enforced by minimising complexity. Crucially, minimising complexity minimises metabolic cost.

In short, the infomax principle can be understood in terms of the decomposition of free energy into complexity and accuracy: mutual information or statistical efficiency is optimised when conditional expectations maximise accuracy (or minimise prediction error), while thermodynamic efficiency is assured by minimising complexity. This minimisation ensures that the generative model is not over-parameterized and leads to a parsimonious representation of sensory data that conforms to prior beliefs about their causes. Interestingly, advanced model optimisation techniques use free-energy optimisation to eliminate redundant model parameters [98], suggesting that free-energy optimisation might provide a nice explanation for synaptic pruning and homeostasis in the brain during neurodevelopment [99] and sleep [100]. In developing the link between metabolic and statistical efficiency, we have assumed that internal neuronal states encode hidden states in terms of their most likely value or expectation. Is there any principled reason to assume this form of neuronal code?

### The Maximum Entropy Principle and the Laplace Assumption

Notice from Equation 7 that minimising variational free energy entails maximising the entropy of the conditional density. Intuitively, this is like keeping one's options open when trying to find hypotheses or explanations for sensory input. If we admit an encoding of the conditional density up to second order moments, then the maximum entropy principle [42], implicit in the definition of free energy, requires 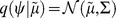 to be Gaussian. This is because a Gaussian density has the maximum entropy of all forms that can be specified with two moments. Assuming a Gaussian form is known as the Laplace assumption and enables us to express the entropy of the conditional density in terms of its first moment or expectation. This follows because we can minimise free energy with respect to the conditional covariance as follows:
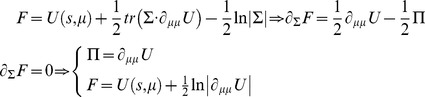
(18)Here, the conditional precision Π(*μ*) is the inverse of the conditional covariance Σ(*μ*). Equation 18 means the free energy becomes a function of conditional expectations and sensory states. This is important because it suggests the brain may represent hidden states of the world in terms of their expected values. This leads to the Laplace code (defined as neuronal encoding under the Laplace assumption), which is arguably the simplest and most flexible of all neuronal codes [13], [14]. Furthermore, under the Laplace code, one can minimise free energy efficiently using predictive coding [29], [31]. Predictive coding has become one of the most popular ways of understanding message passing in the brain—particularly in the setting of hierarchical perceptual inference. In short, the free-energy principle entails the principle of maximum entropy and leads, in a principled way, to a neuronal encoding of representations in terms of conditional expectations.

The specific nature of the neural code may be exclusive to a species or underlying neural function. Whatever its makeup—expected latency, firing rate, spike timing, phase, etc.—it will exist to harmonize the dialogue between perception and action. In practice, we usually have in mind the instantaneous rate of firing of neuronal populations, which means the internal states encoding posterior beliefs are ensemble averages of ensemble averages—for example, the expectation of (a function of) depolarisation over the neuronal ensemble, where the depolarisation of a single neuron is (a function of) the internal variables of a canonical ensemble.

## Conclusion

We have reviewed the thermodynamic and computational (statistical) imperatives for biological self-organisation, with a special focus on neuronal circuits. We have considered the role of classical thermodynamics and the notion of metabolic efficiency—that appears to be an important constraint, under which neurophysiology and neuroanatomy have evolved. From a computational perspective, we have looked at variational free-energy minimisation as the basis for active Bayesian inference and modelling of the environment. The ability to represent and predict hidden environmental states efficiently can be quantified in terms of mutual information. Our synthesis suggests that minimising variational free energy is a sufficient account of the tendency to maximise both metabolic and statistical efficiency. The motivation for minimising variational free energy is to minimise its long-term average to maintain a constant external milieu—as measured by the entropy of an organism's sensory samples over time. By decomposing variational free energy into accuracy and complexity one can understand metabolic efficiency in terms of minimising complexity (which minimises Helmholtz free energy), under the computational constraint that sensory inputs are represented accurately. Conversely, statistical efficiency can be understood in terms of maximising the accuracy (which maximises mutual information), under the constraint that representations have minimal complexity. The link between complexity and metabolic cost rests on the simple observation that, in the absence of sensory input, prior beliefs are encoded by physical variables that minimise Helmholtz free energy.

The nice thing about this formulation is that, under active inference, organisms will selectively sample sensory inputs that conform to their prior beliefs and minimise the complexity of their representations. This means that biological systems will appear to act in a way that minimises fluctuations in Helmholtz free energy—and will aspire to the nonequilibrium steady state that has been assigned to them by evolution.
